# High-Performance NH_3_ Sensing via Humidity-Mediated Proton Conduction in Electrospun Amorphous Sn/Ce-Containing Polymer Membranes

**DOI:** 10.3390/nano16171081

**Published:** 2026-08-31

**Authors:** Yuqing Su, Tieda Jin, Gaoshan Zeng, Mingjia Li, Jiantao Wang, Yi Chen, Yuchao Wang, Yongpeng Zhao, Hui Huang

**Affiliations:** 1 College of Mechanical and Electrical Engineering, Sichuan Agricultural University, Ya’an 625014, China; 202305646@stu.sicau.edu.cn (Y.S.); 202405762@stu.sicau.edu.cn (T.J.); zenggaoshan05@gmail.com (G.Z.); 202204874@stu.sicau.edu.cn (M.L.); 2024317027@stu.sicau.edu.cn (J.W.); 202405832@stu.sicau.edu.cn (Y.C.); wangyc0918@yahoo.co.jp (Y.W.); zhaoyp@sicau.edu.cn (Y.Z.); 2 Smart Agriculture Engineering Technology Center of Sichuan Province, Sichuan Agricultural University, Ya’an 625014, China

**Keywords:** NH_3_ sensing, electrospun nanofibers, amorphous hybrid structure, high-humidity sensing, proton conduction

## Abstract

Precise monitoring of ammonia (NH_3_) in humid agricultural environments is essential for livestock management and environmental protection. However, conventional metal oxide semiconductor sensors often suffer from signal attenuation and baseline instability because of competitive water adsorption under room-temperature, high-humidity conditions. Here, a non-annealed Sn/Ce-containing polyacrylonitrile (PAN) nanofiber membrane was fabricated by electrospinning for room-temperature NH_3_ sensing. The resulting Sn/Ce/PAN membrane exhibits an amorphous hybrid structure formed through interactions between the metal species and the PAN matrix. The Sn/Ce/PAN membrane-based sensor delivers a response of 90% toward 100 ppm NH_3_ at 80% RH, with a response time of 16 s and a recovery time of 37 s. The sensing response increases with relative humidity and reaches its maximum at 80% RH, demonstrating excellent sensing performance under high-humidity conditions. Combined experimental and theoretical investigations reveal that the outstanding sensing performance originates from humidity-mediated proton conduction enabled by enhanced water adsorption and the formation of a continuous hydrogen-bond network, whereas excessive water accumulation suppresses charge transport under excessively humid conditions. This work provides mechanistic insights into room-temperature NH_3_ sensing under high humidity and offers a promising strategy for developing high-performance gas sensors for practical humid environments.

## 1. Introduction

Ammonia accumulation in enclosed livestock facilities poses risks to animal health, production efficiency, and the surrounding environment [1,2,3,4,5]. Reliable NH_3_ monitoring in these environments requires sensors that can operate at room temperature while maintaining stable and reversible responses under the high relative humidity commonly encountered in agricultural settings [6,7]. The sensing performance under such conditions is strongly governed by the composition and microstructure of the active sensing layer. Therefore, rational design of sensing materials that combine accessible adsorption sites, efficient charge transport, and humidity tolerance is essential for reliable room-temperature NH_3_ detection.

Polymer-based sensing materials have attracted considerable attention for room-temperature NH_3_ detection because of their low processing temperature, mechanical flexibility, and tunable chemical functionality [8,9,10,11,12,13]. Electrospinning further provides interconnected nanofiber networks with accessible surfaces and continuous pathways for gas transport. In addition, the amorphous regions of polymer matrices can provide free volume, structurally diverse coordination environments, and accessible interaction sites, which facilitate gas diffusion and adsorption without requiring high-temperature crystallization. Ebadi et al. deposited polyaniline onto electrospun polyurethane nanofibers by in situ polymerization and obtained a porous core–shell sensing layer capable of detecting low concentrations of NH_3_ at room temperature [8]. Radhe Shyam et al. introduced fluorinated and alkyl groups into polythiophene and fabricated an oriented organic transistor sensing layer that retained approximately 85% of its initial response at85% relative humidity (RH) [9]. These studies demonstrate the potential of polymer architectures for low-temperature gas sensing and humidity tolerance. However, their performance often depends strongly on polymer orientation, post-functionalization, fiber morphology, and environmental humidity. Variations in water uptake can also cause baseline drift, incomplete recovery, or unstable gas adsorption. Therefore, a simple amorphous polymer nanofiber membrane that combines efficient NH_3_ transport with controlled water-mediated charge conduction remains desirable.

Instead of treating water only as an interfering species, recent studies have used adsorbed water to establish hydrogen-bonded pathways for proton-assisted charge transport at room temperature. Sonda et al. developed a cerium-modified proton-conducting graphene oxide film combined with a Pt/SnO_2_ sensing electrode and achieved ppm-level CO detection at 25 °C [14]. Zheng et al. constructed an AuAg-decorated sodium titanate material that combined surface proton conduction with internal electronic charge transfer for room-temperature acetonitrile sensing [15]. These reports indicate that water-mediated proton transport can reduce the dependence on thermal activation and improve room-temperature sensing kinetics [16,17,18,19]. Nevertheless, the conductivity of such materials is strongly influenced by the continuity and thickness of the adsorbed water layer. Insufficient hydration interrupts proton-transfer pathways, whereas excessive water accumulation can occupy adsorption sites and hinder target-gas transport [17,20,21,22]. In addition, many reported proton-conducting sensors rely on multistep inorganic heterostructure fabrication and have not been specifically designed for NH_3_ detection within the 70–90% RH range relevant to agricultural environments.

Herein, a non-annealed Sn/Ce-containing polyacrylonitrile (PAN) nanofiber membrane was fabricated by one-step electrospinning for room-temperature NH_3_ sensing under humid conditions. The nitrile groups of PAN serve as coordination sites for Sn and Ce species, while the interconnected fibrous network facilitates the diffusion of NH_3_ and water molecules through the sensing layer. By tuning the Ce/Sn ratio, the coordination environment, NH_3_ adsorption capability, and humidity-dependent charge transport are effectively regulated. Among the investigated compositions, Sn-Ce_30%_ exhibits a linear response over 5–200 ppm NH_3_ with an R^2^ value above 0.999 and delivers a response of 90% toward 100 ppm NH_3_ at 80% RH. The sensing response reaches its maximum at 80% RH, demonstrating excellent sensing performance under high-humidity conditions. Combined experimental and theoretical investigations reveal that the optimized bimetallic coordination environment enhances NH_3_ adsorption and promotes humidity-mediated proton conduction through a continuous hydrogen-bond network. This work provides an effective strategy for developing room-temperature NH_3_ sensors for practical operation in humid environments.

## 2. Materials and Methods

### 2.1. Chemicals and Materials

Polyacrylonitrile (PAN; Mw ≈ 1,000,000) was purchased from Sinopec, Chengdu, China. Stannous chloride dihydrate (SnCl_2_·2H_2_O) was obtained from Chengdu Jinshan Chemical Reagent Co., Ltd., Chengdu China. Cerium(III) nitrate hexahydrate (Ce(NO_3_)_3_·6H_2_O) was purchased from Adamas-beta^®^ (Titan Scientific), Shanghai, China. N,N-Dimethylformamide (DMF, HCON(CH_3_)_2_) was obtained from Chengdu Jinshan Chemical Reagent Co., Ltd., Chengdu China. Deionized water was used for all solution preparation and washing procedures throughout the experiments.

### 2.2. Preparation of Sn/Ce/PAN Composite Nanofiber Membranes

To systematically investigate the influence of dual-metal coordination and metal molar ratios on gas-sensing performance, a series of composite nanofiber networks with different Ce/Sn molar ratios were fabricated via electrospinning (Figure S1, Supporting Information). The sample compositions are summarized in Table S1 (Supporting Information). In a typical synthesis, predetermined amounts of Ce(NO_3_)_3_·6H_2_O and SnCl_2_·2H_2_O with specific molar ratios were accurately weighed and dissolved in 15 mL of DMF under magnetic stirring at 25 °C for 10 min, yielding a clear and homogeneous precursor solution. Subsequently, 2 g of polyacrylonitrile (PAN) powder was slowly added to the solution. The mixture was stirred in a thermostatic water bath at 60 °C for 30 min to facilitate polymer dissolution and then continuously stirred at room temperature for 12 h. A viscous, homogeneous, particle-free, and bubble-free spinning solution was thus obtained. The prepared precursor solution was transferred into a 60 mL disposable syringe equipped with a stainless-steel needle (inner diameter: 0.5 mm). The syringe was mounted on a syringe pump with a feeding rate of 0.4 mL h^−1^. During electrospinning, a high-voltage direct-current power supply was applied with the spinneret connected to the positive electrode and the collector connected to the negative electrode. The applied voltage was maintained at 15 kV, and the tip-to-collector distance was fixed at 15 cm. A rotating metal collector covered with silicone-coated paper was used to collect the electrospun fibers. The electrospinning process was carried out in a closed chamber at 25 °C and a relative humidity of 70%. Under the applied electric field, the precursor jet underwent rapid stretching and thinning accompanied by solvent evaporation, resulting in the formation of continuous composite nanofiber membranes on the collector. The as-spun membranes were dried in a vacuum oven at 60 °C for 4 h to remove residual solvent, yielding the final non-annealed Sn/Ce/PAN nanofiber membranes. The resulting membrane could be removed from the silicone-coated collector paper as a continuous, self-supporting sheet, as shown in Figure S2a,b (Supporting Information).

### 2.3. Characterization

The morphologies of the samples were characterized using field-emission scanning electron microscopy (FESEM, Hitachi SU8020, Tokyo, Japan), scanning electron microscopy (SEM, ZEM 18, ZEPTOOLS, Tongling, China), and transmission electron microscopy (TEM, JEOL JEM-2100, Tokyo, Japan). The crystal structure was investigated by X-ray diffraction (XRD, Anton Paar XRDynamic500, AT) over a 2θ range from 10° to 80°. The chemical bonding characteristics were analyzed using Fourier transform infrared spectroscopy (FTIR, Thermo Scientific Nicolet iS20, USA). The elemental composition and surface chemical states were determined using X-ray photoelectron spectroscopy (XPS, Thermo Scientific K-Alpha, USA).

### 2.4. Assembly and Testing of Gas Sensor Devices

The fabricated devices are two-terminal chemiresistive NH_3_ sensors based on interdigitated electrodes. The sensing signal is transduced as a change in the electrical resistance of the Sn/Ce/PAN nanofiber membrane upon NH_3_ exposure. The non-woven nanofiber membranes were cut into 3 mm × 3 mm rectangular pieces with a thickness of approximately 10 μm. Each membrane was mounted on a ceramic substrate equipped with interdigitated electrodes with an electrode gap of 0.5 mm. Conductive copper-foil adhesive was used to establish electrical contact between the nanofiber membrane and the electrodes. The assembled devices were connected to a WS-30B gas-sensing test system (Zhengzhou Weisheng Electronic Technology Co., Ltd., Zhengzhou China) for resistance measurements. Photographs of the sensor fabrication and assembly process and the actual WS-30B measurement setup are provided in Figure S2c,d of the Supporting Information. A DC bias voltage of 5 V was applied during the sensing measurements, and the resistance signal was acquired at a rate of 1 measurement s^−1^. The test chamber had an effective volume of 18 L. The experiment used analytically pure ammonia water as the gas source. The concentration of the target gas was calculated by the formula [23]:

(1) $$V=\frac{PV_{0}CM}{RT\rho },$$

*V*_0_ represents the volume of the test chamber, *P* is the standard atmospheric pressure, *M* is the molar mass of the target gas, *R* is the gas constant, *T* is the environmental temperature, and *ρ* is the density of the target gas. The required injection volume (*V*) is determined based on the chamber volume (*V*_0_ = 18,000 mL), the target gas concentration (*c*), the molecular weight of ammonia (*M* = 17 g/mol), the density of the ammonia solution (d = 0.91 g/mL), and its purity (*p* = 25%). Based on these parameters, 6 µL of ammonia solution is required to achieve an ammonia concentration of 100 ppm. This volume is precisely drawn using a micro-syringe and rapidly injected onto a heated platform, where it instantly vaporizes. The resulting vapor is then uniformly mixed with the air inside the chamber by a fan, yielding the target gas at the predetermined concentration. Thus, a gradient of specific target concentrations of 5, 20, 50, 80, 100, 150, and 200 ppm can be obtained. Relative humidity was controlled by accurately withdrawing deionized water with a microsyringe and rapidly injecting it onto a heated platform inside the sensing chamber, where the water was rapidly vaporized. An internal fan was used to facilitate the dispersion and uniform mixing of the generated water vapor. Before each measurement, the chamber was allowed to equilibrate for approximately 5 min until the sensing-system signal reached a stable state. The RH was monitored using a sensor positioned inside the chamber below the sensing device, with an accuracy of ±2% RH. During NH_3_ exposure, the RH remained essentially unchanged, indicating negligible humidity variation during the sensing measurement. The response value of the sensor is uniformly defined as:

(2) $$Response\left(\%\right)=\frac{R_{a}-R_{g}}{R_{g}}\times 100\%,$$

Here, *R_a_* represents the initial baseline resistance of the sensor under a specific relative humidity air background, while *R_g_* is the real-time resistance when the target gas reaches a steady state. Additionally, the response time and recovery time are defined as the time required for the resistance change to reach 90% of the total change amplitude. All gas sensitivity tests were repeated three times at room temperature (20 °C) to ensure the reproducibility of the data.

### 2.5. Theoretical Calculations

Density functional theory (DFT) calculations were performed using the Quantum ESPRESSO package within the generalized gradient approximation (GGA) using the Perdew-Burke-Ernzerhof (PBE) functional. A finite PAN short-chain model coordinated with Sn and Ce species was constructed to investigate the interactions among the Sn/Ce/PAN framework, NH_3_, and H_2_O molecules. All calculations were carried out in a sufficiently large cubic supercell with Γ-point sampling to eliminate interactions between periodic images. The atomic structures were fully optimized using the BFGS algorithm until the total energy and forces reached the convergence criteria. Based on the optimized structures, adsorption energies, differential charge density, Bader charge, and proton migration pathways were calculated. The proton transfer energy barrier was determined using the nudged elastic band (NEB) method. Detailed computational parameters, including pseudopotentials, plane-wave cutoff energies, convergence criteria, and NEB settings, are provided in the Supporting Information.

## 3. Results and Discussion

### 3.1. Material Structure and Microscopic Morphology Characterization Analysis

**Figure 1:**
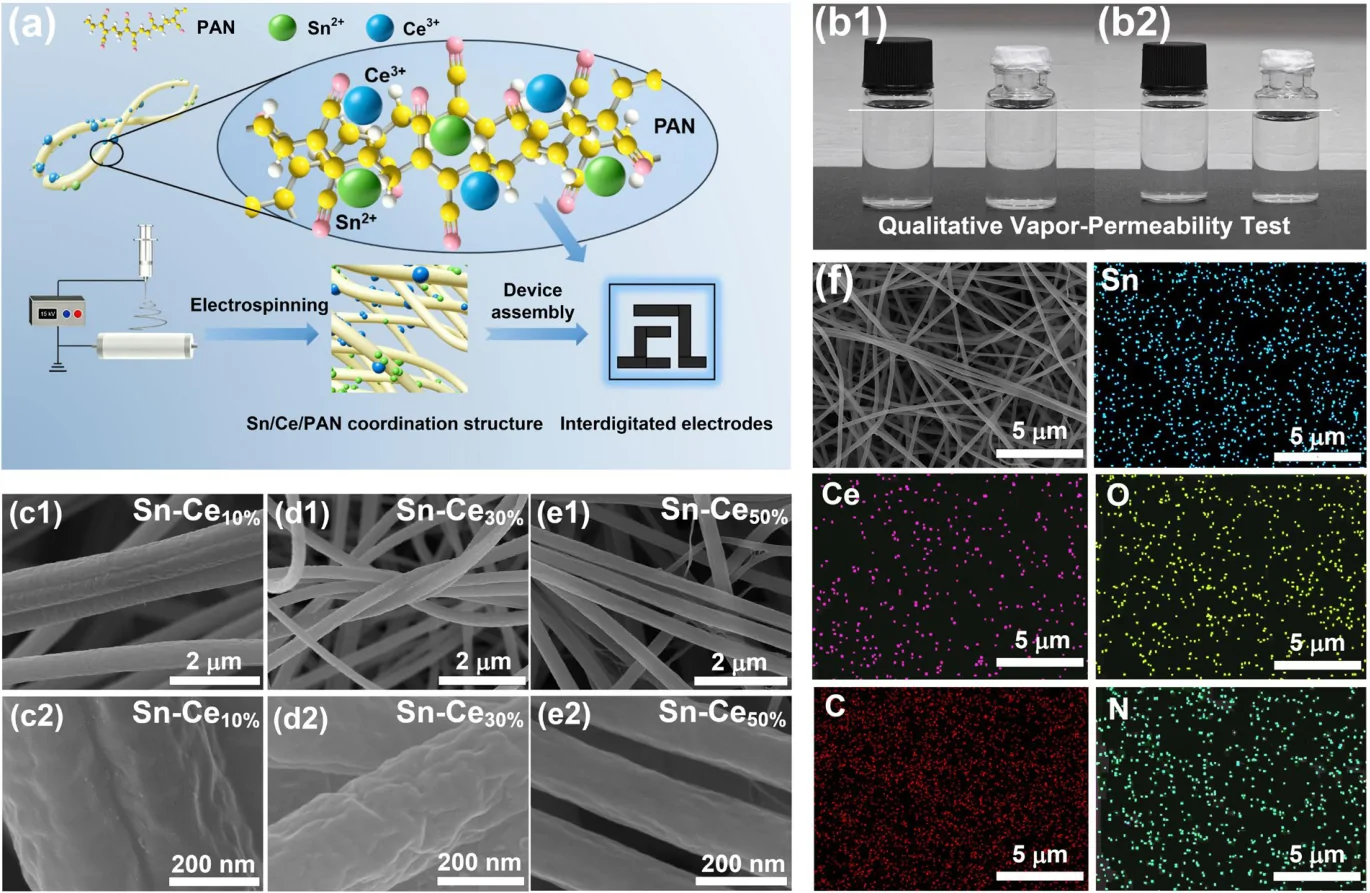
(**a**) Schematic diagram of the fabrication of the Sn/Ce/PAN composite nanofiber membrane sensor; (**b1**,**b2**) Qualitative vapor-permeability test over three days. (**b1**) Initial liquid levels in a sealed control vial and a vial covered with the nanofiber membrane. (**b2**) Corresponding liquid levels after three days; (**c1**,**c2**) SEM images of Sn-Ce_10%_ at various magnifications; (**d1**,**d2**) SEM images of Sn-Ce_30%_ at various magnifications; (**e1**,**e2**) SEM images of Sn-Ce_30%_ at various magnifications; (**f**) EDS elemental mapping.

For room-temperature NH_3_ sensing under humid conditions, an effective sensing membrane should provide continuous pathways for gas and water transport, accessible interaction sites, and a structurally continuous network for stable resistance modulation. Accordingly, the morphology, structural state, and elemental distribution of the non-annealed Sn/Ce/PAN nanofiber membranes were systematically examined to clarify how the Ce/Sn ratio regulates these characteristics. Figure 1a schematically illustrates the fabrication process of the non-annealed Sn/Ce-coordinated PAN composite nanofiber membrane via a one-step electrospinning technique. As illustrated, the Sn and Ce species are incorporated into the PAN matrix through coordination interactions with the nitrile (-C≡N) groups along the polymer chains. The vapor permeability of the non-annealed Sn/Ce/PAN composite nanofiber membrane was qualitatively evaluated using a sealed vial as the control, as shown in Figure 1(b1,b2). At the initial stage, the liquid levels in the sealed control vial and the vial covered with the nanofiber membrane were comparable (Figure 1(b1)). After three days, the liquid level in the sealed control vial remained nearly unchanged, whereas a distinct decrease was observed in the membrane-covered vial (Figure 1(b2)). Meanwhile, the nanofiber membrane remained intact. These observations indicate that water vapor can permeate through the interconnected pores of the fibrous membrane. Such a permeable network may facilitate the transport of water vapor and NH_3_ molecules into and through the sensing layer, thereby improving their accessibility to the internal sensing interfaces.

The SEM images shown in Figure 1c–e reveal that the Ce/Sn molar ratio has a significant influence on the fiber morphology. At a Ce/Sn ratio of 1:9 (Figure 1(c1,c2)), the Sn-Ce_10%_ membrane consists of a dense, randomly interconnected network of ultrafine nanofibers with continuous and bead-free morphology. Owing to the dominant presence of the Sn precursor, the high solution conductivity facilitates pronounced jet stretching under the applied electric field, leading to the formation of uniform nanofibers with an average diameter of 815 nm. When the Ce/Sn molar ratio was increased to 3:7 (Figure 1(d1,d2)), the Sn-Ce_30%_ membrane exhibited a relatively uniform and open three-dimensional fibrous network with larger inter-fiber voids than Sn-Ce_10%_. The fibers presented a highly clear, uniform, and spatially open three-dimensional cross-linked non-woven mesh framework. While the high-magnification SEM image reveals a more pronounced wrinkled and undulating surface morphology, this feature is likely associated with changes in the solidification, and the pore distribution between the fibers exhibited outstanding spatial connectivity. Meanwhile, as shown in Figure S3 (Supporting Information), the Sn-Ce_30%_ composite nanofiber membrane exhibits a characteristic amorphous hybrid morphology without discernible aggregation of crystalline metal oxide particles on the fiber surface. Such a hierarchical porous architecture, together with the wrinkled fiber surface, is expected to increase the accessible surface area and facilitate the adsorption of water molecules as well as the diffusion of NH_3_ molecules to the active sensing sites, thereby promoting gas-sensing performance.

When the Ce/Sn molar ratio is further increased to 1:1 (Figure 1e), the Sn-Ce_50%_ nanofiber morphology deteriorates markedly. The continuity of the fibrous network decreases, while pronounced local fiber swelling, inter-fiber adhesion, and partial fiber fusion are observed. Compared with Sn-Ce_30%_, the Sn-Ce_50%_ membrane shows a noticeably lower degree of surface wrinkling, and the fiber surfaces become relatively smoother. This morphological evolution is likely associated with changes in the rheological and solidification behavior of the spinning solution at the higher Ce/Sn ratio. The altered metal-polymer interactions and solvent evaporation kinetics may reduce the differential shrinkage between the rapidly solidified surface layer and the fiber interior, thereby suppressing surface buckling and wrinkle formation. At the same time, the increased tendency toward local plasticization and incomplete fiber solidification promotes adhesion and fusion between adjacent fibers. The EDS elemental mapping of Sn-Ce_30%_ (Figure 1f) reveals a homogeneous distribution of C, O, Ce, and Sn throughout the three-dimensional nanofiber network, indicating the uniform incorporation of Ce and Sn species within the PAN matrix. This result suggests that the metal ions are well dispersed through coordination interactions with the nitrile (-C≡N) groups of PAN, preventing localized aggregation during the electrospinning process.

**Figure 2:**
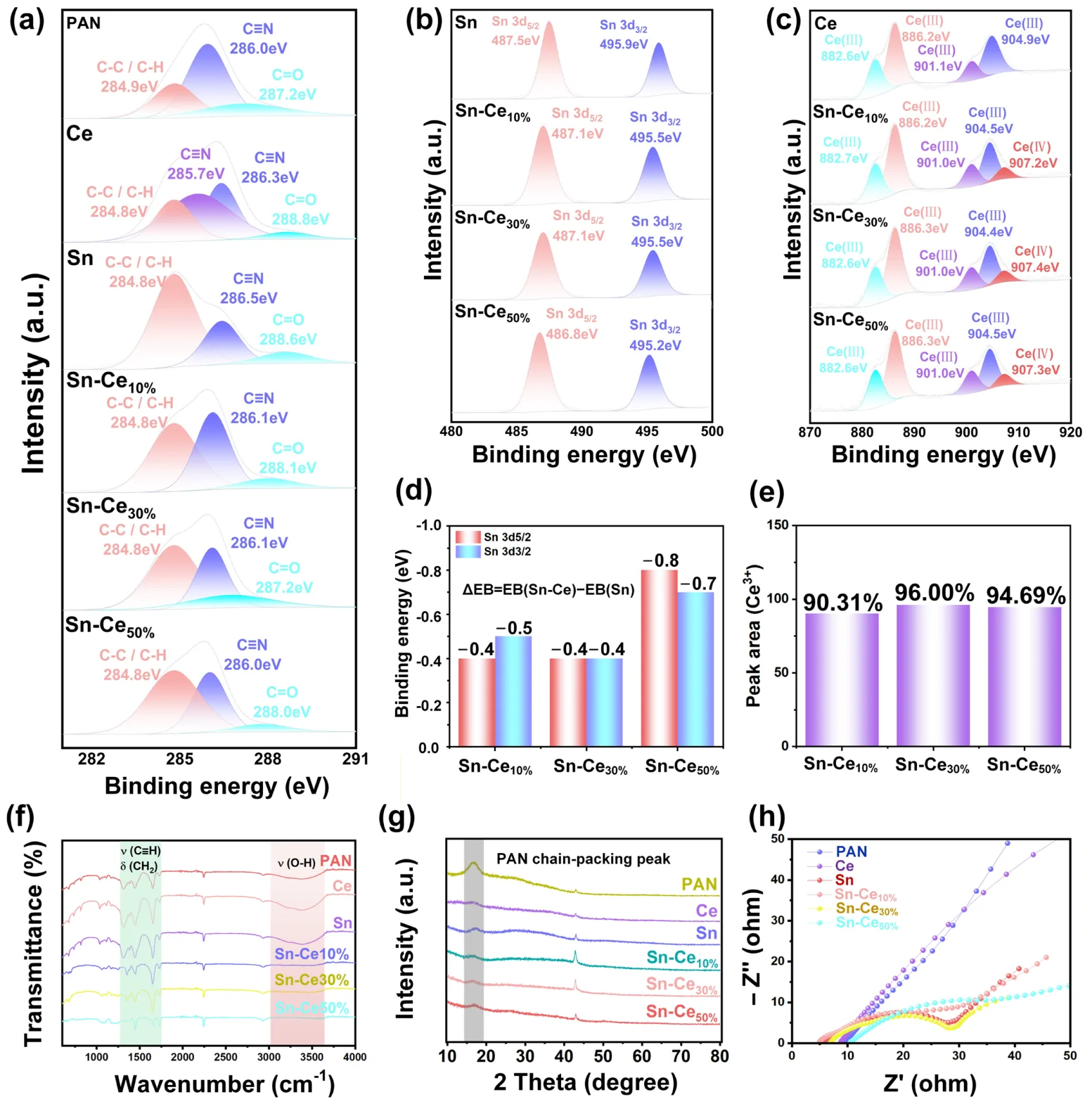
High-resolution (**a**) C 1s, (**b**) Sn 3d, and (**c**) Ce 3d XPS spectra of the Sn/Ce/PAN composite nanofiber membrane; (**d**) Binding-energy shifts in the Sn 3d_5_/_2_ and Sn 3d_3_/_2_ peaks of Sn-Ce_10%_, Sn-Ce_30%_, and Sn-Ce_50%_ relative to the corresponding Sn 3d peaks of the Sn sample located at 487.5 and 495.9 eV, respectively; (**e**) Ratio of Ce^3+^ to the total Ce species (Ce^3+^ + Ce^4+^); (**f**) FTIR spectra; (**g**) XRD patterns; and (**h**) Nyquist plots.

The surface chemical states and electronic interactions within the sensing materials were investigated by XPS. As shown in Figure 2a, the C 1s spectrum exhibits two characteristic peaks at approximately 284.8 and 286.0 eV, corresponding to C-C/C-H and C≡N bonds, respectively. A slight shift in the C≡N peak is observed after the introduction of metal species, indicating the coordination interaction between the nitrile groups of PAN and the metal cations through lone-pair electron donation, leading to the formation of stable coordination bonds [24]. The high-resolution Sn 3d spectra are shown in Figure 2b. For the pristine Sn sample, the Sn 3d_5_/_2_ and Sn 3d_3_/_2_ peaks are located at approximately 487.5 and 495.5 eV, respectively. With increasing Ce incorporation (Sn-Ce_10%_, Sn-Ce_30%_, and Sn-Ce_50%_), both peaks gradually shift toward lower binding energies, reaching 486.8 and 495.2 eV for Sn-Ce_50%_. These negative shifts indicate electron redistribution between Ce and Sn, confirming strong interfacial electronic coupling and charge transfer between the two metal species [25]. The Ce 3d spectra (Figure 2c) display the characteristic multiplet splitting of mixed Ce^3+^/Ce^4+^ states, confirming the coexistence of the two valence states. Quantitative peak fitting (Figure 2e) reveals Ce^3+^/(Ce^3+^ + Ce^4+^) ratios of 90.31%, 96.00%, and 94.69% for Sn-Ce_10%_, Sn-Ce_30%_, and Sn-Ce_50%_, respectively [26]. Among them, Sn-Ce_30%_ exhibits the highest relative Ce^3+^ content, suggesting a distinct local redox and coordination environment around the Ce-containing sites. The interfacial electronic redistribution between the Sn- and Ce-containing species may promote local charge polarization within the nanofiber network. This modified local electronic environment may favor the adsorption of NH_3_ and H_2_O and contribute to the enhanced sensing response of Sn-Ce_30%_ [27,28,29].

FTIR spectroscopy was employed to further investigate the molecular interactions within the composite nanofibers (Figure 2f). All samples exhibit a sharp absorption band at 2242 cm^−1^, corresponding to the characteristic stretching vibration of the nitrile (C≡N) groups in PAN [30,31]. Compared with pristine PAN, slight variations in the vibration bands at 1452 cm^−1^ and 1360 cm^−1^, assigned to the CH_2_ bending and CH vibrations, respectively, are observed after metal incorporation, suggesting interactions between the polymer chains and the coordinated metal species [32]. A pronounced difference is observed in the 3000–3660 cm^−1^ region, which is attributed to the stretching vibration of hydroxyl groups and hydrogen-bonded water molecules [33,34,35,36]. Pristine PAN exhibits only a weak absorption in this region, reflecting its limited affinity for water molecules. After introducing Sn or Ce, the broad O-H absorption band becomes evident, while the Sn-Ce_30%_ sample exhibits the highest peak intensity and the largest integrated area. These results indicate that dual-metal coordination effectively enhances surface hydroxylation and promotes the formation of an extensive hydrogen-bond network on the nanofiber surface. The enriched surface hydroxyl groups provide abundant adsorption sites for water molecules, which is expected to facilitate NH_3_ adsorption and subsequent sensing reactions, thereby contributing to the superior humidity-tolerant gas-sensing performance of the Sn-Ce_30%_ sensor. XRD patterns (Figure 2g) show a broad diffraction peak centered at 2θ ≈ 17° for all samples, which is assigned to the microcrystalline domains of the PAN polymer chains [37]. No characteristic diffraction peaks corresponding to crystalline SnO or CeO_2_ are observed throughout the measured range, indicating that the incorporated metal species remain highly dispersed within the PAN matrix without forming detectable crystalline oxide phases [38]. This result suggests that the metal species are incorporated into the polymer as an amorphous inorganic phase. Compared with their crystalline counterparts, the amorphous structure is expected to possess a higher density of structural defects and free volume, which is beneficial for NH_3_ adsorption and charge transport during the gas-sensing process [39].

Impedance spectroscopy was used to compare the electrical transport characteristics of the nanofiber membranes over the frequency range of 0.1 Hz to 99.95 kHz (Figure 2h). Because the spectra were not fitted using an equivalent circuit, the apparent series resistance (R_s,app_) was estimated from the minimum Z’ value in the high-frequency region above 10 kHz. The R_s,app_ values of PAN, Ce, Sn, Sn-Ce_10%_, Sn-Ce_30%_, and Sn-Ce_50%_ are approximately 9.141, 8.795, 4.839, 16.970, 7.002, and 5.813 Ω, respectively. These values show that Sn-Ce_30%_ does not possess the lowest high-frequency series resistance. More pronounced differences are observed in the low-frequency region. At 0.1 Hz, the impedance magnitudes of PAN, Ce, Sn, Sn-Ce_10%_, Sn-Ce_30%_, and Sn-Ce_50%_ are 1055.0, 580.6, 50.27, 156.80, 40.47, and 44.67 Ω, respectively. Sn-Ce_30%_ therefore exhibits the lowest low-frequency impedance among the representative curves, followed by Sn-Ce_50%_ and Sn, whereas PAN and Ce show substantially higher impedance values. Linear fitting of the low-frequency branches over 0.10–1.00 Hz gives slopes of 0.476, 0.857, 0.753, 0.788, 0.805, and 1.140 for PAN, Ce, Sn, Sn-Ce_10%_, Sn-Ce_30%_, and Sn-Ce_50%_, respectively. The lower low-frequency impedance of Sn-Ce_30%_ indicates more facile interfacial charge transport under the measurement conditions, consistent with its enhanced NH_3_-sensing response.

### 3.2. Gas Sensing Performance Test

**Figure 3:**
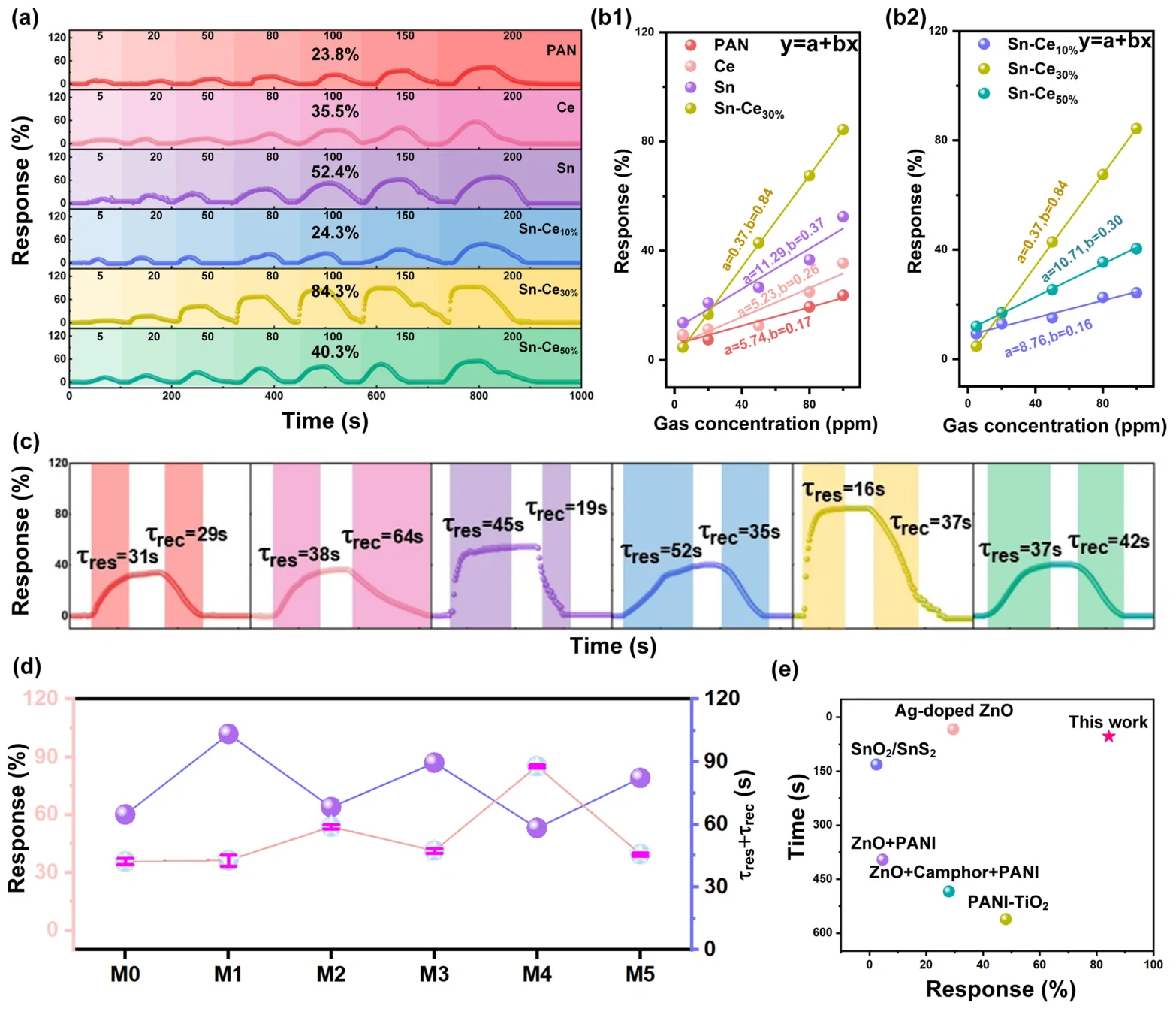
(**a**) Dynamic response curves of different sensors toward various NH_3_ concentrations. (**b1**,**b2**) Corresponding calibration curves. (**c**) Response and recovery curves of different sensors. (**d**) Response values and response/recovery times of different sensors. (**e**) Comparison of the NH_3_ sensing performance of this work with previously reported sensors.

Test results indicate that the various nanofiber membranes exhibit distinct charge-transport characteristics. However, these electrical differences alone are insufficient to determine their gas-sensing performance. Consequently, the NH_3_-sensing behavior of this series of sensors was subsequently evaluated under identical temperature and humidity conditions, with a focus on concentration-dependent response, reversibility, and response-recovery kinetics. Figure 3 shows the dynamic response-recovery behavior of the sensors toward 5, 20, 50, 80, 100, 150, and 200 ppm NH_3_ at room temperature. Reproducibility and baseline recovery are important criteria for evaluating the practical applicability of gas sensors. Figure S4 (Supporting Information) presents the continuous cyclic response-recovery curves of the Sn-Ce_30%_ sensor toward NH_3_ concentrations from 5 to 200 ppm at 20 °C and 70% RH. The results indicate that the response increases with increasing NH_3_ concentration. After each air-purge step, the signal returns close to the initial baseline, indicating reversible and repeatable sensing behavior under the investigated humid conditions.

As shown in Figure 3a, the Sn-Ce_30%_ sensor exhibits a pronounced stepwise concentration-dependent response. As the NH_3_ concentration increases from 5 to 200 ppm, the response of the Sn-Ce_30%_ sensor increases progressively, and the baseline resistance returns close to its initial level after each air-purge step. At 100 ppm NH_3_, the representative responses of PAN, Ce, Sn, Sn-Ce_10%_, Sn-Ce_30%_, and Sn-Ce_50%_ are 23.8%, 35.5%, 52.4%, 24.3%, 84.3%, and 40.3%, respectively. The response of Sn-Ce_30%_ is approximately 4.6 times that of Sn, which exhibits the second-highest response in the representative dynamic curves. As shown in Figure 3b, the calibration curve of Sn-Ce_30%_ exhibits good linearity over the investigated concentration range, with a correlation coefficient (R^2^) greater than 0.999. The fitted slope of Sn-Ce_30%_ is 6.15% ppm^−1^, which is significantly higher than those of the other sensors. Based on the standard deviation of the blank response and the fitted calibration slope, the limit of detection (LOD) of the Sn-Ce_30%_ sensor is calculated to be 1 ppm using LOD = 3σ/k. To quantify the effects of metal incorporation and Ce/Sn ratio on concentration sensitivity, linear fitting and quantitative analysis were performed for PAN, Ce, Sn, Sn-Ce_10%_, Sn-Ce_30%_, and Sn-Ce_50%_ (Figure S5, Supporting Information). The corresponding slopes are 0.17, 0.26, 0.37, 0.16, 0.84, and 0.30 ppm^−1^, respectively. The slope of Sn-Ce_30%_ is approximately 2 times that of Sn and more than 2.5 times those of Sn-Ce_10%_ and Sn-Ce_50%_. Comparison of the fitted slopes and R^2^ values indicates that Sn-Ce_30%_ exhibits the strongest concentration-dependent response among the investigated sensors. These results identify Sn-Ce_30%_ as the best-performing composition among the tested bimetallic ratios.

In addition to response magnitude, response and recovery times are important parameters for evaluating gas-sensing performance, as shown in Figure 3c. The response-recovery curves are presented using two *y*-axis ranges because the response of Sn-Ce_30%_ is substantially higher than those of the other samples. The response/recovery times of PAN, Ce, Sn, Sn-Ce_10%_, Sn-Ce_30%_, and Sn-Ce_50%_ are 31/29, 38/64, 45/19, 52/35, 16/37, and 37/42 s [40], respectively. Sn-Ce_30%_ exhibits the shortest response time of 16 s, whereas Sn exhibits the shortest recovery time of 19 s. The total response-recovery duration of Sn-Ce_30%_ is 53 s, compared with 60, 102, 64, 87, and 79 s for PAN, Ce, Sn, Sn-Ce_10%_, and Sn-Ce_50%_, respectively. The relatively rapid response may be associated with the open and interconnected structure of the non-annealed Sn/Ce/PAN nanofiber membrane, which provides accessible pathways for NH_3_ transport through the sensing layer. Figure 3d compares the response values and total response-recovery times of the sensors at 100 ppm NH_3_. Considering the response magnitude, calibration slope, linearity, and response-recovery kinetics, Sn-Ce_30%_ exhibits the best overall performance among the investigated sensors.

The room-temperature NH_3_-sensing performance of Sn-Ce_30%_ was further compared with representative sensors reported previously (Figure 3e). For example, the SnO_2_/SnS_2_ sensor exhibited a response of 2.48% with a response time of 131 s toward 100 ppm NH_3_, while the PANI/TiO_2_ sensor showed a response of 48% and a response time of 561 s under comparable conditions. In comparison, the Sn-Ce_30%_ sensor exhibits a response of 90% toward 100 ppm NH_3_, together with a response time of 16 s. Thus, while its response kinetics are comparable to those of the representative high-performance sensors, its response magnitude is substantially higher. Overall, Sn-Ce_30%_ achieves a favorable balance between response intensity and response speed among the room-temperature NH_3_ sensors considered in Figure 3e [41,42,43,44].

**Figure 4:**
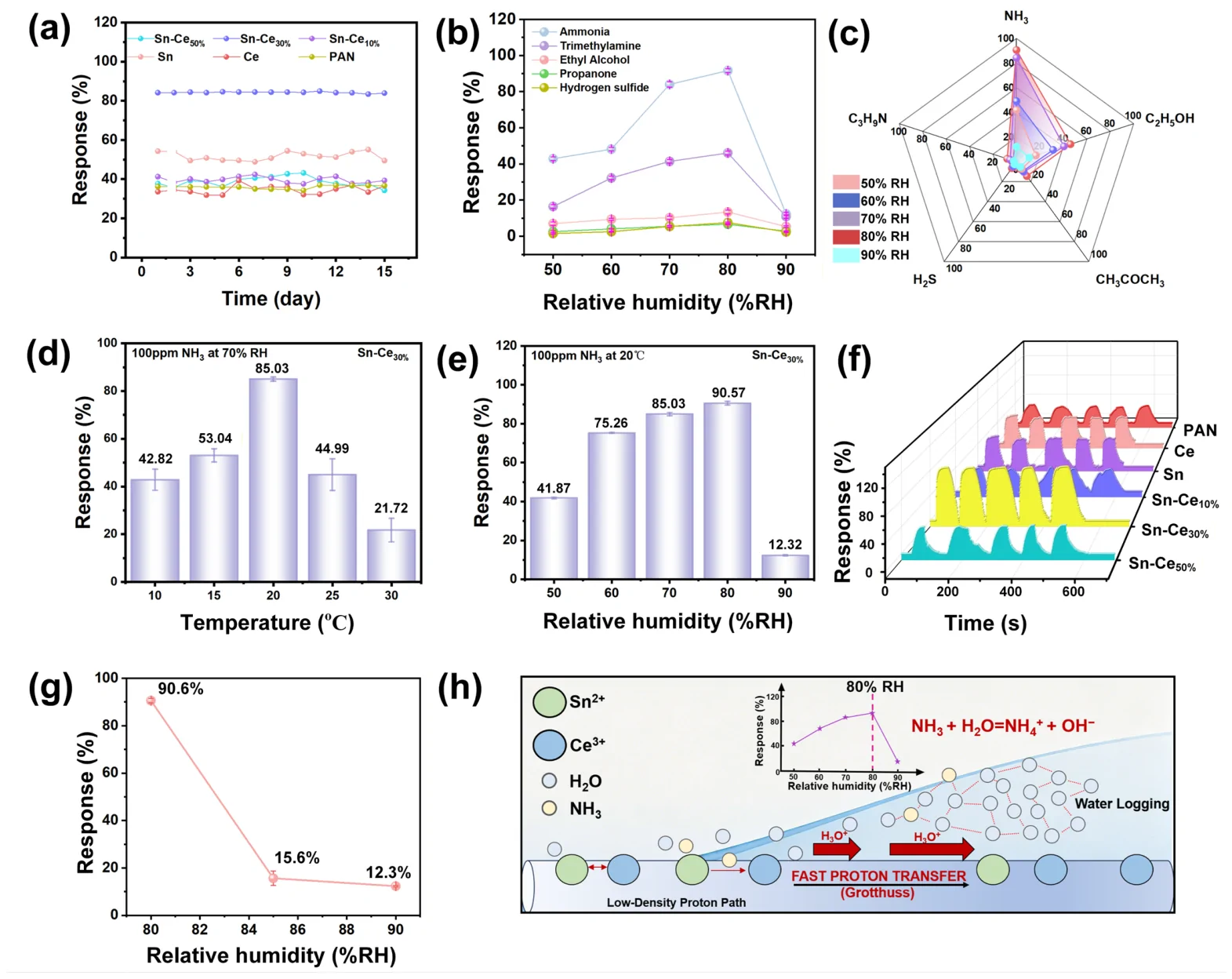
(**a**) Long-term stability of the sensors toward 100 ppm NH_3_ at 20 °C and 70% RH. (**b**) Responses of the Sn-Ce_30%_ sensor to 100 ppm NH_3_ and 100 ppm interfering gases under different RH conditions. (**c**) Radar-chart representation of the sensing performance. (**d**) Response of Sn-Ce_30%_ to 100 ppm NH_3_ at different temperatures (70% RH). (**e**) Response of Sn-Ce_30%_ to 100 ppm NH_3_ at different RH levels (20 °C). (**f**) Cycling stability of different sensors. (**g**) Response of Sn-Ce_30%_ to 100 ppm NH_3_ over an RH range of 80–90% at 20 °C. (**h**) Proposed NH_3_ sensing mechanism of the Sn/Ce composite nanofibers under different RH conditions.

To comprehensively evaluate the feasibility of the Sn/Ce/PAN composite nanofiber membrane sensor for practical environmental monitoring, especially in high-humidity scenarios such as livestock farming, its gas-sensing performance was systematically investigated. As shown in Figure 4a, the response of Sn-Ce_30%_ toward 100 ppm NH_3_ remains nearly constant at approximately 84.3% throughout a 15-day stability test, demonstrating excellent long-term stability. Consistently, the cyclic response curves (Figure 4f) exhibit negligible signal attenuation or baseline drift, confirming the excellent operational stability and fatigue resistance of the sensor. The gas selectivity of Sn-Ce_30%_ was further evaluated using 100 ppm NH_3_ and 100 ppm of each interfering gas, including trimethylamine, ethanol, acetone, and hydrogen sulfide, under different relative humidity (RH) conditions (Figure 4b). The NH_3_ response increases from 41.87% at 50% RH to 75.26%, 85.03%, and 90.57% at 60%, 70%, and 80% RH, respectively. In contrast, the responses to the interfering gases remain substantially lower over the same humidity range. At 80% RH, the response to NH_3_ increases sharply to 90.57%, whereas the responses to (CH_3_)_3_N, C_2_H_5_OH, CH_3_COCH_3_, and H_2_S remain substantially lower. This result indicates enhanced NH_3_ selectivity at 80% RH. The corresponding radar chart (Figure 4c) further highlights the exceptional NH_3_ selectivity under high-humidity conditions. These results indicate that water molecules function not as interfering species but as a key synergistic component that promotes the selective sensing of NH_3_. The combined effects of temperature and humidity were further investigated. As shown in Figure 4d, at 70% RH, the response toward 100 ppm NH_3_ reaches 85.03% at 20 °C, indicating optimal sensing performance at room temperature. Figure 4e further shows that, at 20 °C, the response increases with RH and reaches a maximum value of 90.57% at 80% RH. When the RH is further increased to 85% and 90%, however, the response decreases sharply to 15.6% and 12.3%, respectively (Figure 4g). To further evaluate the influence of environmental conditions on NH_3_ sensing, the response of the Sn-Ce_30%_ sensor toward 100 ppm NH_3_ was systematically investigated over a combined temperature range of 10–30 °C and RH range of 50–90%. The complete response matrix is provided in Figures S6–S10 (Supporting Information). At a fixed temperature, the NH_3_ response generally increases with RH from 50% to 80% RH, reaches a maximum within the intermediate-to-high humidity region, and then decreases markedly at 90% RH. The temperature dependence also varies with RH, indicating that temperature and humidity jointly regulate the sensing response. Under the tested conditions, the highest response is observed at 20 °C and 80% RH, whereas the response decreases substantially at 80 °C and 90% RH. These results demonstrate that the sensor response is strongly dependent on both temperature and humidity and identify a favorable operating window under the investigated conditions. The systematic temperature dependence of the chemiresistive response highlights the potential for multifunctional sensing. Recently, InSe-based sensing platforms have demonstrated dual-mode sensing capabilities, enabling simultaneous detection of gas concentration and temperature within a single system and highlighting the potential of multiparameter sensing for environmental monitoring. Related studies have further demonstrated the integration of gas and temperature sensing functions in flexible MWCNTs–InSe devices using independent sensing channels and system-level monitoring capabilities [45]. In contrast, the Sn-Ce_30%_ device investigated in the present study currently provides a single chemiresistive output and was developed primarily for NH_3_ detection. Consequently, the systematic temperature dependence observed here provides a basis for future integration of an independent temperature-sensitive channel or a calibrated temperature-sensing element to enable simultaneous monitoring of NH_3_ and temperature. Additionally, the mechanical deformation tolerance of the Sn-Ce_30%_ sensor was further evaluated to assess its potential for flexible sensing applications (Figure S11, Supporting Information). Figure S11a–d present photographs of the Sn-Ce_30%_ nanofiber membrane in rolled, stretched, folded, and twisted states. As shown in Figure S11e–h, clear and reproducible NH_3_ response–recovery characteristics were maintained at bending angles of 0°, 45°, and 90°. The average response values were 84.0% at 0°, 81.2% at 45°, and 78.6% at 90°, corresponding to response retentions of 96.7% and 93.6% at 45° and 90°, respectively, relative to the response at 0°. of the initial response, respectively. These results indicate that the applied mechanical deformation caused only minor changes in the sensing response, and the sensing functionality of the Sn-Ce_30%_ nanofiber membrane was largely retained under bending. Combined with the self-supporting film morphology, these results support the potential for integration into flexible sensing platforms, while further evaluation on flexible substrates and under repeated mechanical cycling is warranted.

### 3.3. High-Humidity Sensing Mechanism

The pronounced humidity dependence of the NH_3_ response indicates that adsorbed water plays an important role in the sensing process. At 50–70% RH, the relatively limited amount of adsorbed water may result in insufficient hydrogen-bond connectivity for efficient proton-assisted charge transport. As the RH increases to 80%, enhanced water adsorption at the Sn/Ce-coordinated sites may promote the formation of more continuous hydrogen-bonded pathways. NH_3_ adsorption at the hydrated interface can perturb the local acid–base equilibrium involving NH_3_/NH_4_^+^ and H_2_O/H_3_O^+^ species. Proton transfer within the hydrogen-bond network may then contribute to the enhanced interfacial charge transport. Such proton transfer can proceed through Grotthuss-type hopping and may also involve vehicular transport of hydrated proton carriers. At 85–90% RH, the sharp decrease in response despite the higher water content suggests that excessive water accumulation may hinder NH_3_ diffusion, occupy adsorption sites, and reduce the measurable resistance modulation. These observations suggest that an intermediate humidity level provides a more favorable balance between water-assisted charge transport and NH_3_ accessibility.

**Figure 5:**
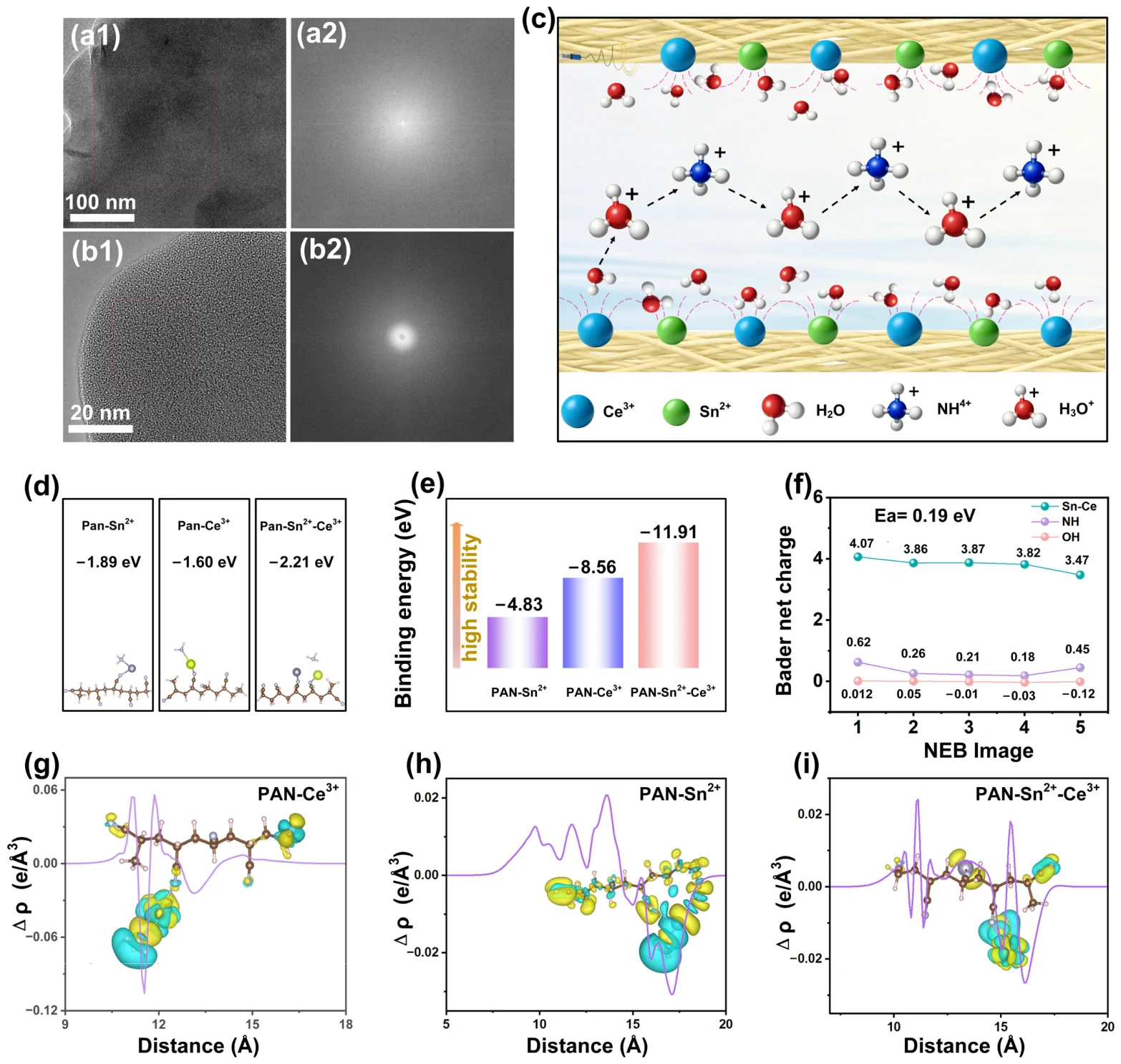
(**a1**,**b1**) TEM and high-resolution TEM images of Sn-Ce_30%_; (**a2**,**b2**) corresponding FFT patterns obtained from the red-boxed regions in (**a1**) and (**b1**), respectively. (**c**) Schematic diagram of the ammonia sensing mechanism of this sensor. (**d**) NH_3_ adsorption energies on the three sensing materials. (**e**) Binding energies of PAN-Sn^2+^, PAN-Ce^3+^, and PAN-Sn^2+^-Ce^3+^. (**f**) Bader net charges of the three model systems. (**g**–**i**) Charge density difference maps of (**g**) PAN-Ce^3+^, (**h**) PAN-Sn^2+^, and (**i**) PAN-Sn^2+^-Ce^3+^.

To examine the local structural state of the Sn-Ce_30%_ nanofibers, transmission electron microscopy was performed. The TEM and high-resolution TEM images in Figure 5(a1,b1) show a continuous nanofiber matrix without discernible lattice fringes or aggregated crystalline particles. The corresponding fast Fourier transform (FFT) patterns obtained from the red-boxed regions are shown in Figure 5(a2,b2). Both FFT patterns display diffuse intensity distributions without distinct diffraction spots, which is consistent with a predominantly amorphous local structure. Combined with the XRD and elemental-mapping results, these observations suggest that the Sn- and Ce-containing species are dispersed within the amorphous PAN matrix without forming detectable crystalline oxide domains.

The interactions between the metal-containing sites and the PAN matrix were first evaluated by calculating the binding energies. As shown in Figure 5e, the binding energies of PAN-Sn^2+^, PAN-Ce^3+^, and PAN-Sn^2+^-Ce^3+^ are −4.83, −8.56, and −11.91 eV, respectively. According to the adopted energy definition, the more negative binding energy of PAN-Sn^2+^-Ce^3+^ indicates a more favorable interaction between the bimetallic center and the nitrile groups of PAN. The enhanced coordination stability may facilitate the retention of the metal-containing sites within the polymer matrix and is consistent with the stable sensing response observed during the 15-day test. The calculated NH_3_ adsorption energies on PAN-Sn^2+^, PAN-Ce^3+^, and PAN-Sn^2+^-Ce^3+^ are −1.89, −1.60, and −2.21 eV, respectively (Figure 5d). Among the investigated configurations, the bimetallic model exhibits the most negative adsorption energy, indicating the strongest calculated affinity toward NH_3_.

Differential charge-density analysis was further performed for the PAN-Ce^3+^, PAN-Sn^2+^, and PAN-Sn^2+^-Ce^3+^ models. As shown in Figure 5g–i, charge accumulation and depletion occur around the metal centers and the neighboring PAN groups, indicating metal-induced redistribution of the local electron density. In PAN-Ce^3+^, the redistribution is mainly localized near the Ce-containing coordination site. PAN-Sn^2+^ displays a broader redistribution involving the Sn center and adjacent polymer segments. For PAN-Sn^2+^-Ce^3+^, both metal centers and the coordinated nitrile groups participate in the charge redistribution, suggesting cooperative electronic interactions within the bimetallic coordination environment. Such redistribution can strengthen the interaction between NH_3_ and the adsorption site and promote interfacial charge variation during gas exposure. The calculated electronic structure is therefore consistent with the higher NH_3_ response of the bimetallic sample compared with the single-metal samples.

The water-mediated proton-transfer pathway was subsequently investigated using the nudged elastic band method. The calculated minimum-energy pathway contains five images and exhibits an activation barrier of 0.19 eV, with the highest-energy configuration located at Image 3 (Figure 5f and Figure S12). The Bader charge of the N-containing group changes from +0.62 e in Image 1 to +0.26, +0.21, and +0.18 e in Images 2–4, respectively, and then increases to +0.45 e in Image 5. This evolution reflects continuous charge redistribution during proton migration between the N-containing group and the adjacent water molecule. Meanwhile, the net charge of the Sn-Ce coordination center decreases from +4.07 to +3.47 e along the pathway, indicating that the bimetallic environment participates in stabilizing the evolving proton-transfer configuration. The OH-containing group maintains a relatively small charge variation of 0.01 to −0.12 e. The low activation barrier and continuous charge redistribution indicate that water-mediated proton transfer is kinetically accessible in the hydrated PAN-Sn^2+^-Ce^3+^ model at room temperature. This process provides an atomic-scale explanation for the enhanced humidity-dependent NH_3_ response of the Sn-Ce_30%_ sensor.

Based on the above structural characterization and DFT calculations, a humidity-mediated proton-conduction mechanism is proposed to explain the outstanding room-temperature NH_3_ sensing performance of the Sn/Ce/PAN composite nanofibers (Figure 5c). Under 80% RH, the synergistic effect of the Sn/Ce bimetallic centers generates strong local electrostatic fields, promoting water adsorption on the membrane surface and facilitating the formation of a continuous hydrogen-bond network. Upon adsorption at the active sites with a high adsorption energy of −2.21 eV, NH_3_ reacts with the adsorbed water layer according to NH_3_ + H_2_O → NH_4_^+^ + OH^−^. The humidity-dependent response and the calculated low-energy-barrier proton transfer pathway indicate that humidity-mediated proton-assisted processes and ion transport contribute to this phenomenon. Proton-assisted charge transport at the hydrated interface may involve Grotthuss-type proton hopping through the hydrogen-bond network, together with vehicular transport of hydrated ionic species such as NH_4_^+^ and H_3_O^+^, thereby markedly accelerating interfacial charge transport. This efficient humidity-mediated proton-conduction pathway accounts for the outstanding room-temperature sensitivity and rapid response/recovery characteristics of the sensor. These findings provide mechanistic insights into humidity-assisted NH_3_ sensing and highlight the effectiveness of dual-metal coordination in regulating water adsorption and proton transport, offering a promising strategy for designing high-performance room-temperature gas sensors for operation under humid conditions.

To further evaluate the practical applicability of the Sn-Ce_30%_ sensor, sensing performance was systematically assessed under environmental conditions representative of agricultural monitoring scenarios. The response to common interfering gases, including trimethylamine, ethanol, acetone, and H_2_S, was evaluated across various humidity levels. The results demonstrated substantially higher responses to NH_3_ than to the selected interfering gases, indicating good selectivity under humid conditions. Overall, effective NH_3_ sensing was maintained under the investigated environmental variations relevant to agricultural applications. At the system level, the chemiresistive Sn-Ce_30%_ device could be integrated with compact temperature and humidity sensors, low-power resistance-acquisition circuitry, and wireless communication modules to form portable or remote monitoring nodes. Such an integrated system could enable simultaneous acquisition of NH_3_ concentration and environmental parameters, while the measured temperature and humidity could be used for environmental compensation of the NH_3_ response. Furthermore, wireless transmission could support remote monitoring of NH_3_ concentrations in enclosed livestock facilities through smartphone- or cloud-based platforms.

## 4. Conclusions

This work presents a non-annealed bimetal-coordination strategy for room-temperature NH_3_ sensing under humid conditions. An electrospun Sn/Ce/PAN composite nanofiber membrane with an interconnected amorphous hybrid structure was developed by regulating the Ce/Sn ratio. Among the investigated compositions, Sn-Ce_30%_ exhibited the best overall sensing performance. The sensor showed a linear response toward 5–200 ppm NH_3_ with an R^2^ value above 0.999 and reached a response of 90.57% toward 100 ppm NH_3_ at 80% RH. It also exhibited reversible response-recovery behavior and stable performance during the 15-day test. The sensing response showed a pronounced dependence on humidity. An appropriate amount of adsorbed water promoted NH_3_ interaction and water-mediated proton transfer, whereas excessive water accumulation at higher humidity suppressed the measurable response. The cooperative Sn/Ce coordination environment further enhanced NH_3_ adsorption and facilitated charge redistribution and proton-transfer processes. These findings demonstrate that the regulation of metal coordination and interfacial water provides an effective strategy for developing room-temperature NH_3_ sensors for humid agricultural environments.

## Data Availability

The data presented in this study are available on request from the corresponding author.

## Supplementary Materials

The following supporting information can be downloaded at: https://www.mdpi.com/article/10.3390/nano16171081/s1, Figure S1: Material preparation process; Figure S2: Photographs showing the fabrication, assembly, and measurement setup of the Sn/Ce/PAN nanofiber membrane sensor. (a) Membrane partially peeled from the silicone-coated collector paper and (b) freestanding membrane. (c1) Membrane mounted on the interdigitated electrode substrate. (c2–c4) Assembled sensor and sensor installation configuration. (d) WS-30B gas-sensing test system and the corresponding data-acquisition interface; Figure S3: SEM images and EDS spectrum of the Sn-Ce_30%_ nanofiber membrane; Figure S4: Cyclic test curves of the Sn-Ce30% sensor under different ammonia concentrations; Figure S5: The linear fitting process: (a) PAN; (b) Ce; (c) Sn; (d) Sn-Ce_10%_; (e) Sn-Ce_30%_; (f) Sn-Ce_50%_; Figures S6–S10: Response values of the Sn-Ce sensor under different humidity conditions; Figure S11: Mechanical deformation and NH_3_-sensing performance of the Sn-Ce_30%_ nanofiber membrane sensor under different bending conditions. (a–d) Photographs of representative mechanical deformations, including rolling, stretching, folding, and twisting, respectively. (e1,f1,g1) Dynamic response–recovery curves toward 100 ppm NH_3_ at bending angles of 0°, 45°, and 90°, respectively. (e2,f2,g2) Corresponding photographs of the sensor under the respective deformation conditions. (h) Average NH_3_ responses of the sensor at different bending angles; Figure S12: Bader net charges of (a) selected atoms and (b) selected molecular groups along the five-image water-mediated proton-transfer pathway in the hydrated PAN-Sn^2+^-Ce^3+^ model; Table S1: The composition of the sample.
