# Supplementary material for: High-Performance NH3 Sensing via Humidity-Mediated Proton Conduction in Electrospun Amorphous Sn/Ce-Containing Polymer Membranes
Source: Nanomaterials (Basel). 2026 Aug 31;16(17):1081. doi: 10.3390/nano16171081 (PMC13567666; doi:10.3390/nano16171081)
Supplement: Supplementary file 1 [file nanomaterials-16-01081-s001.zip › nanomaterials-4539955-supplementary.pdf]

Supporting Information

Article

High-Performance NH_3 Sensing via Humidity-Mediated Proton Conduction in Electrospun Amorphous Sn/Ce-Containing Polymer Membranes

Yuqing Su [1, #], Tieda Jin [1,#], Gaoshan Zeng[1], Mingjia Li [1], Jiantao Wang [1], Yi Chen [1], Yuchao Wang [1, 2], Yongpeng Zhao [1, 2] and Hui Huang [1, 2*]

1 College of Mechanical and Electrical Engineering, Sichuan Agricultural University, Ya'an 625014, PR China

2 Smart Agriculture Engineering Technology Center of Sichuan Province, Sichuan Agricultural University, Ya'an 625014, PR China

# These authors contributed equally to this work.

* Correspondence: huanghui@sicau.edu.cn (H. Huang)

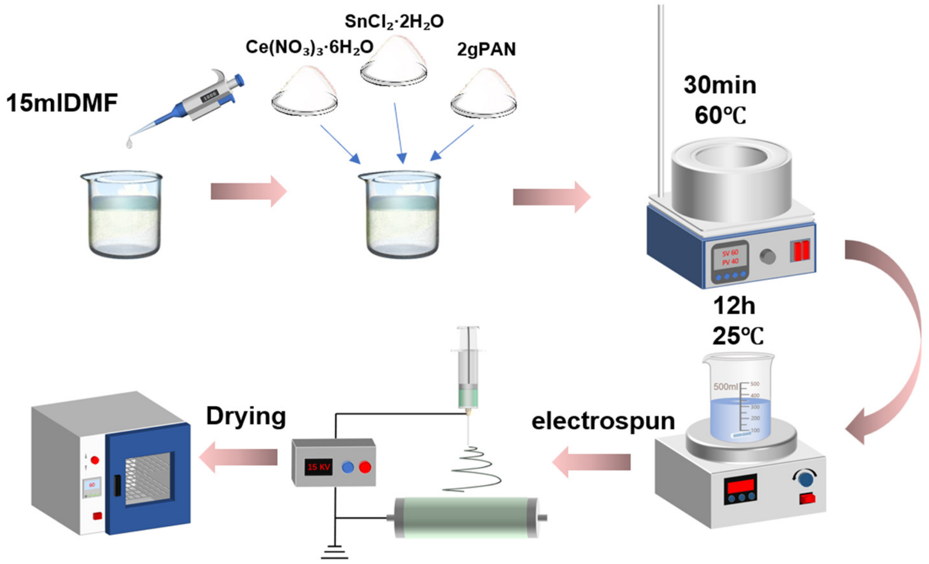

Figure S1. Material preparation process.

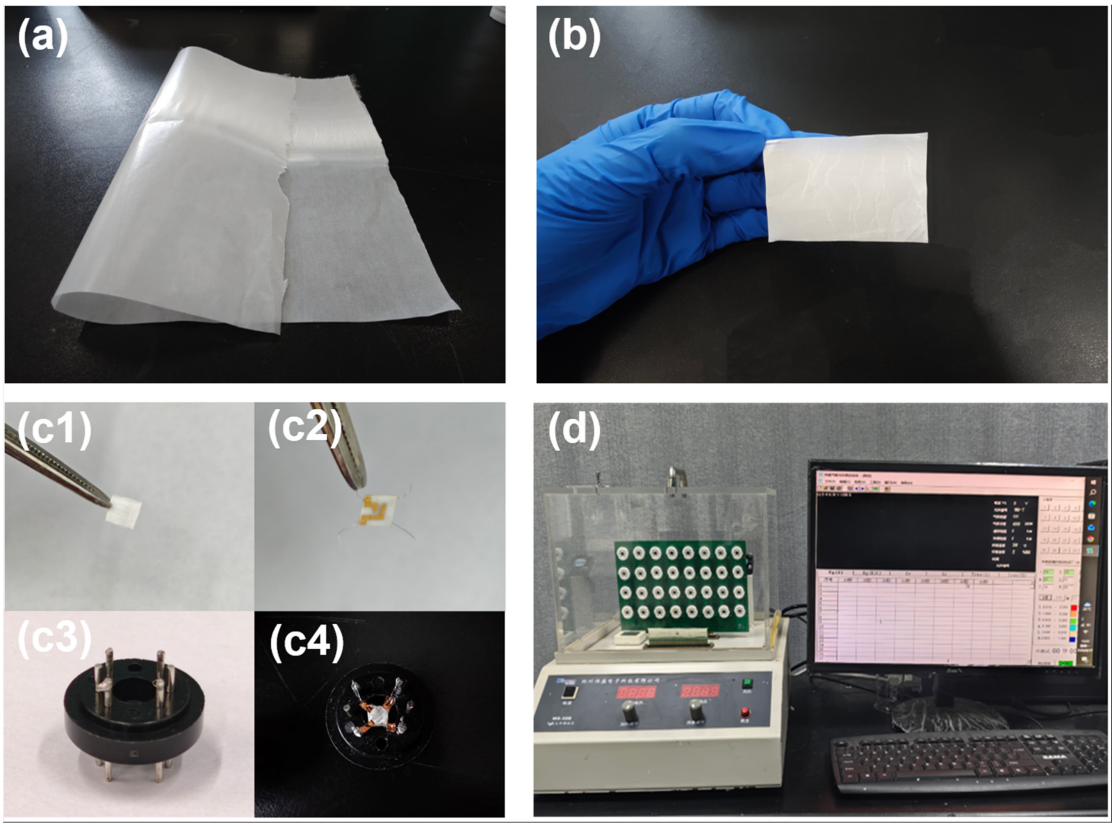

Figure S2. Photographs showing the fabrication, assembly, and measurement setup of the Sn/Ce/PAN nanofiber membrane sensor. (a) Membrane partially peeled from the silicone-coated collector paper and (b) freestanding membrane. (c1) Membrane mounted on the interdigitated electrode substrate. (c2–c4) Assembled sensor and sensor installation configuration. (d) WS-30B gas-sensing test system and the corresponding data-acquisition interface.

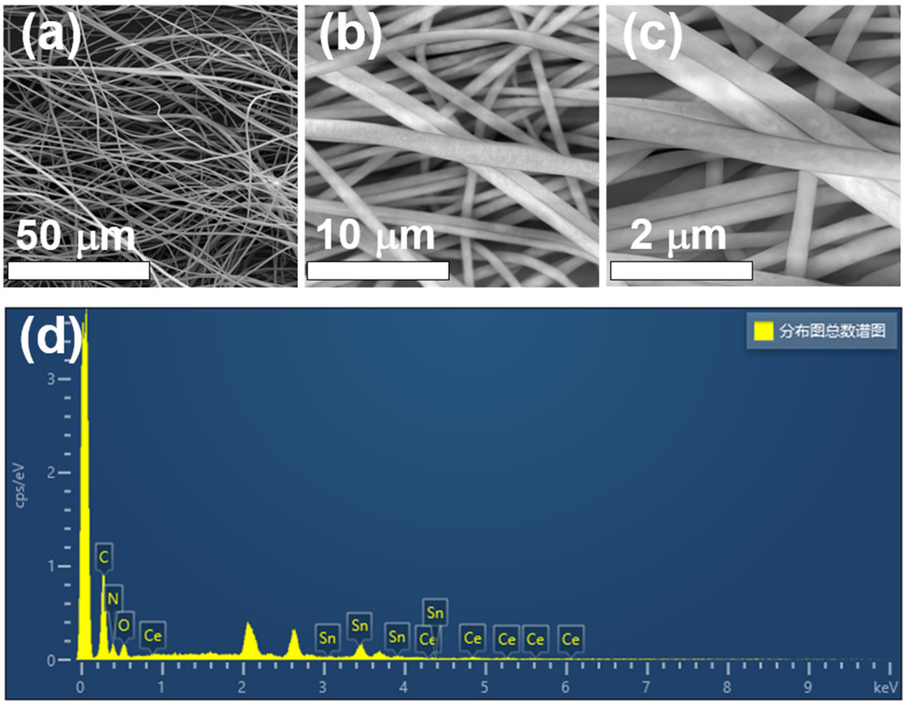

Figure S3. SEM images and EDS spectrum of the Sn-Ce30% nanofiber membrane.

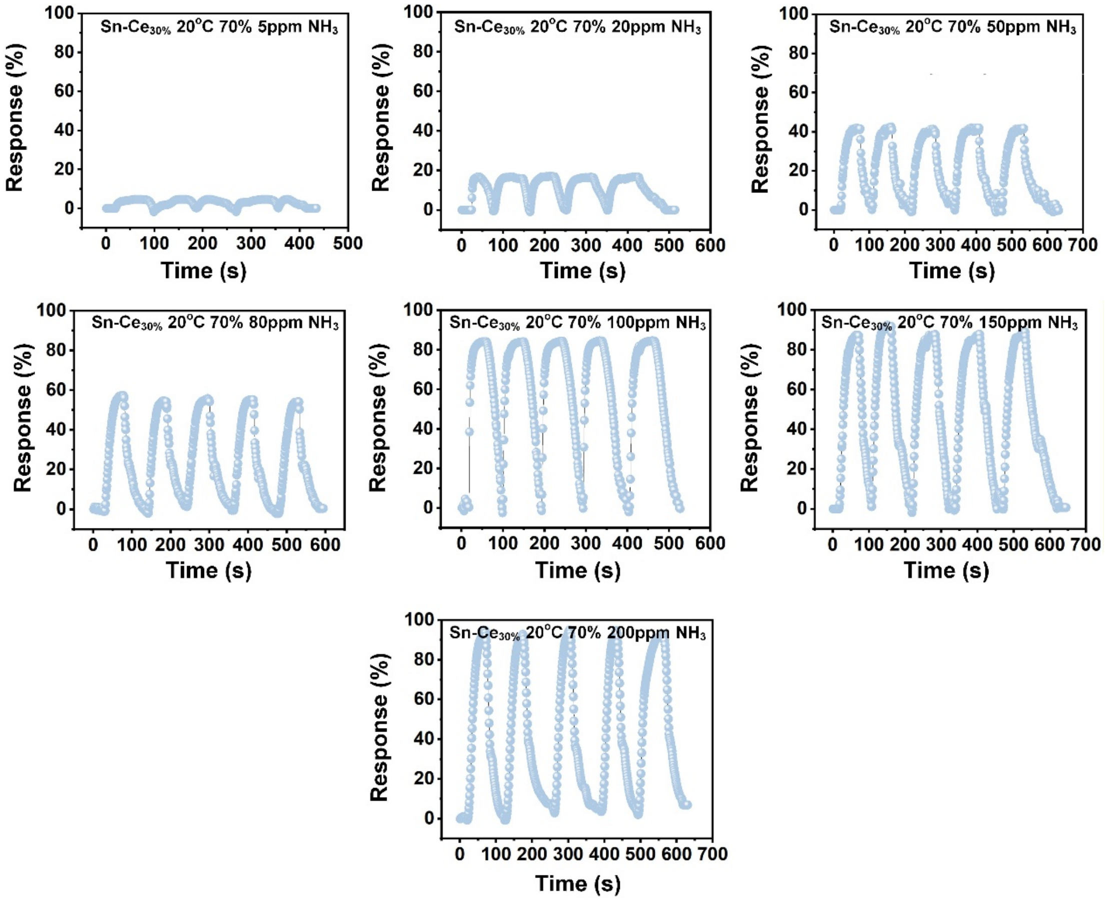

Figure S4. Cyclic test curves of the Sn-Ce30% sensor under different ammonia concentrations.

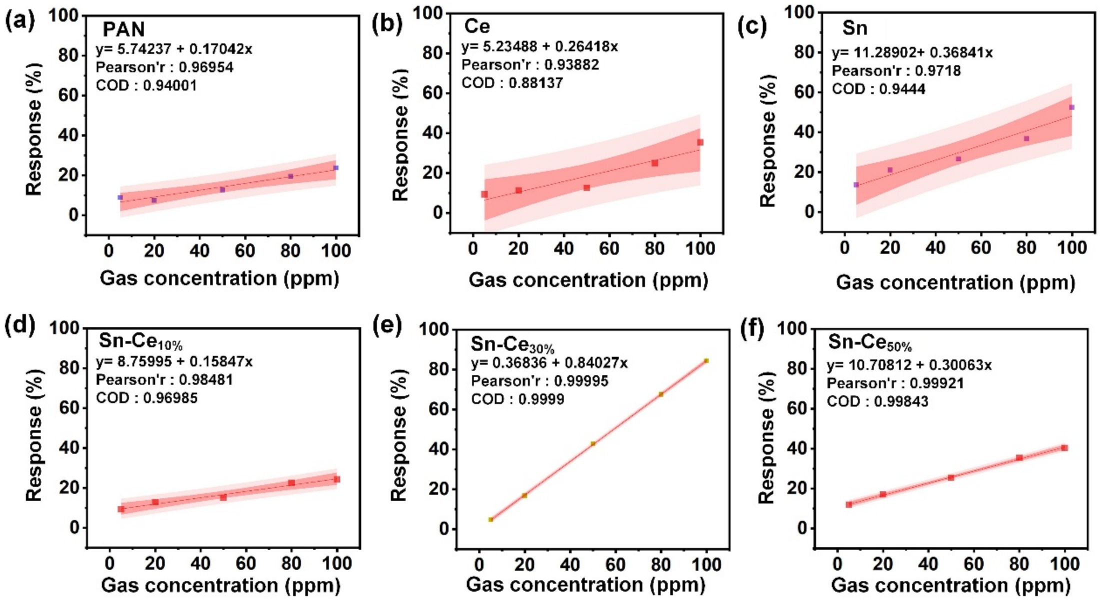

Figure S5. The linear fitting process: (a) PAN; (b) Ce; (c) Sn; (d) Sn-$Ce_{10\%}$; (e) Sn-$Ce_{30\%}$; (f) Sn-$Ce_{50\%}$;

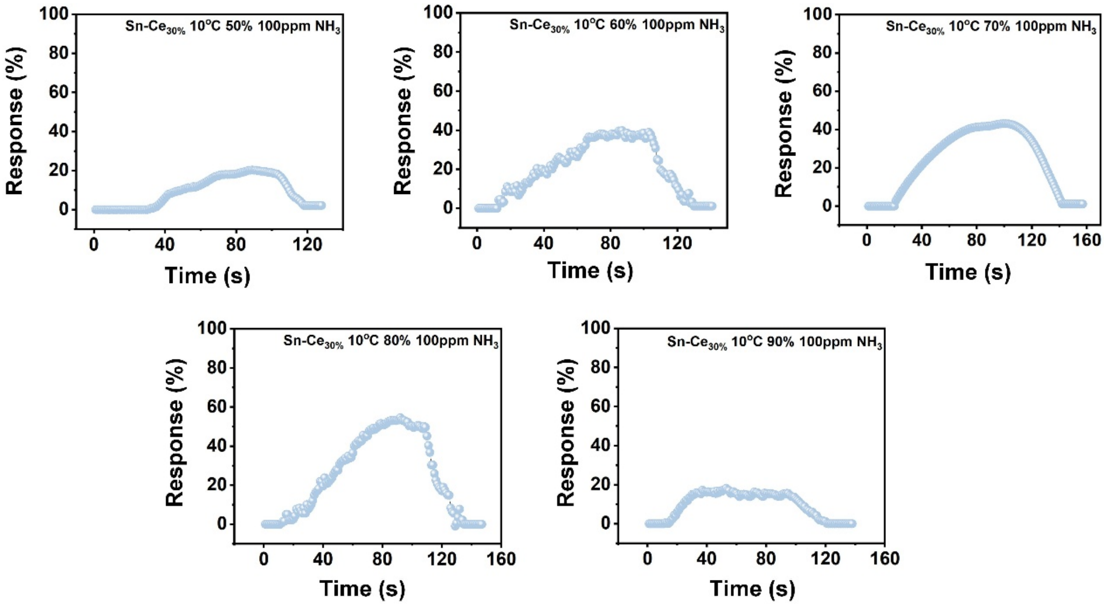

Figure S6. Response values of the Sn-Ce sensor under different humidity conditions.

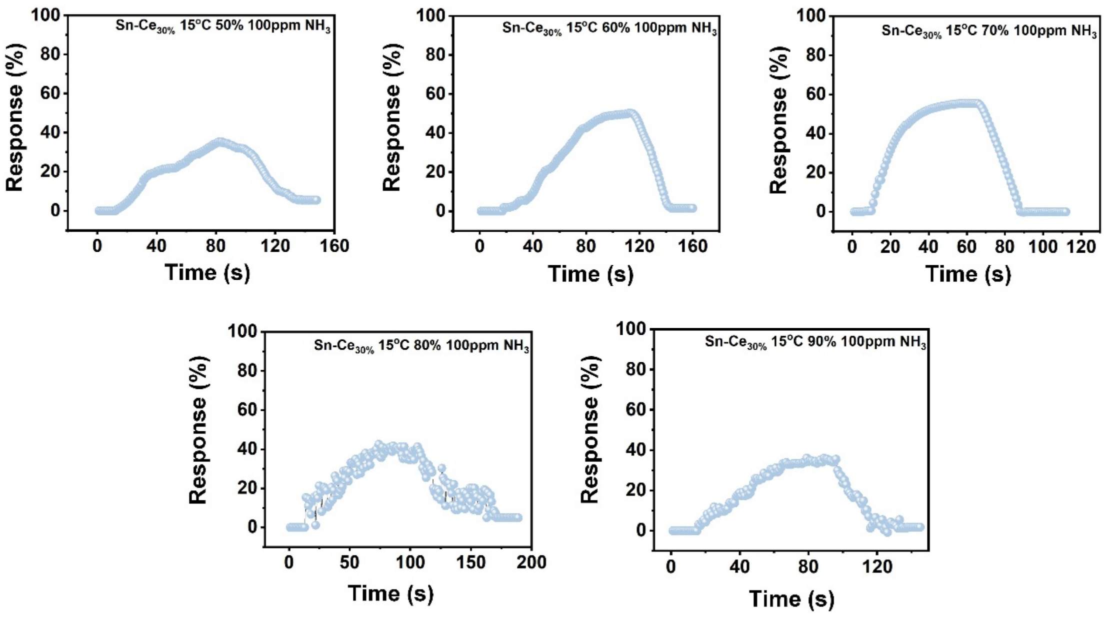

Figure S7. Response values of the Sn-Ce sensor under different humidity conditions.

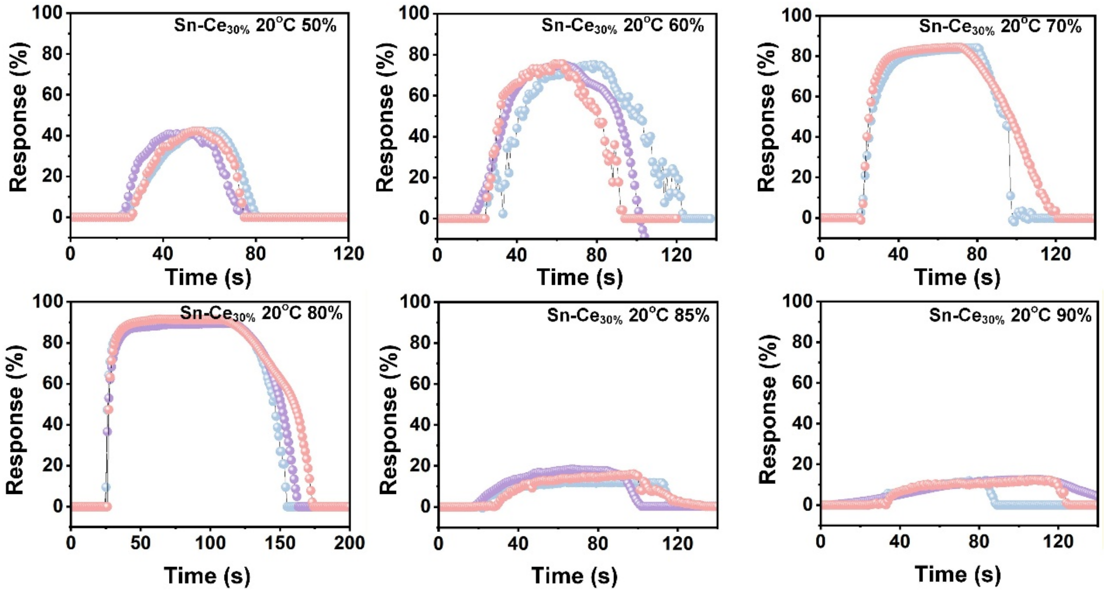

Figure S8. Response values of the Sn-Ce sensor under different humidity conditions.

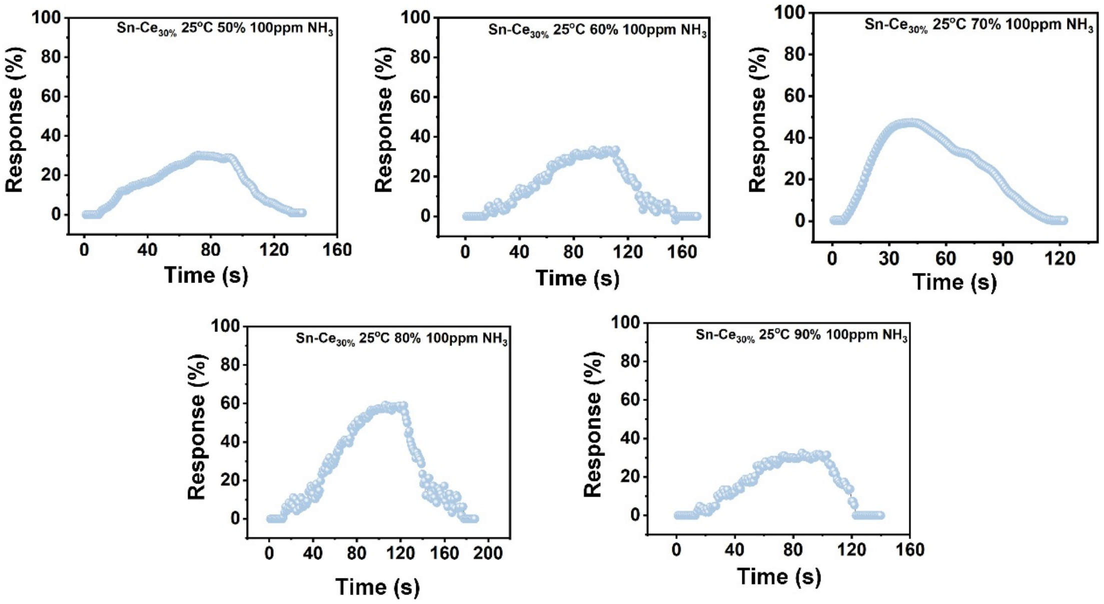

Figure S9. Response values of the Sn-Ce sensor under different humidity conditions.

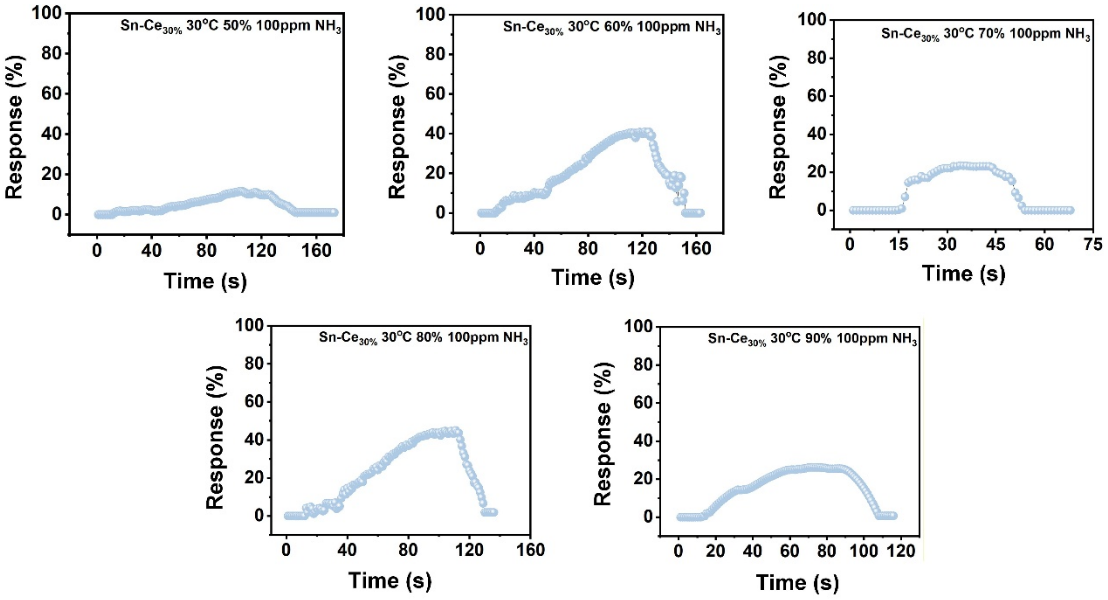

Figure S10. Response values of the Sn-Ce sensor under different humidity conditions.

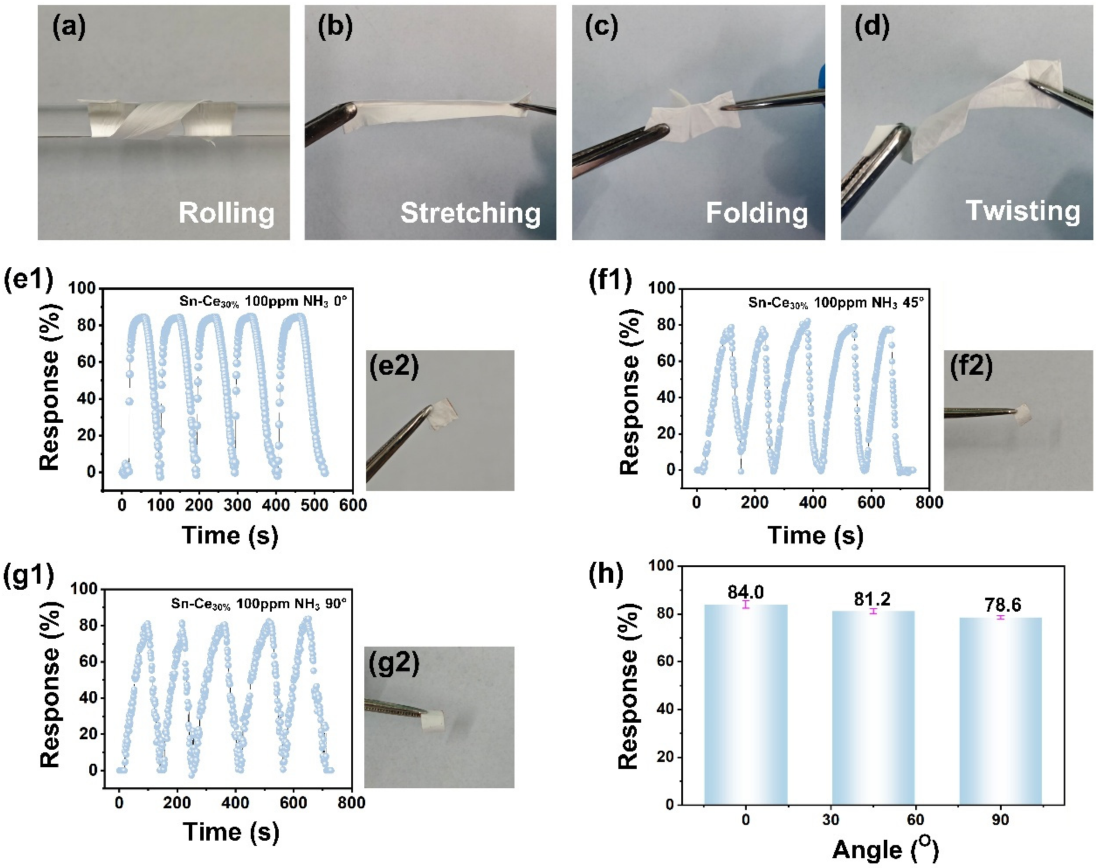

Figure S11. Mechanical deformation and NH_3-sensing performance of the Sn-Ce$_{30\%}$ nanofiber membrane sensor under different bending conditions. (a–d) Photographs of representative mechanical deformations, including rolling, stretching, folding, and twisting, respectively. (e1, f1, g1) Dynamic response–recovery curves toward 100 ppm NH_3 at bending angles of 0°, 45°, and 90°, respectively. (e2, f2, g2) Corresponding photographs of the sensor under the respective deformation conditions. (h) Average NH_3 responses of the sensor at different bending angles.

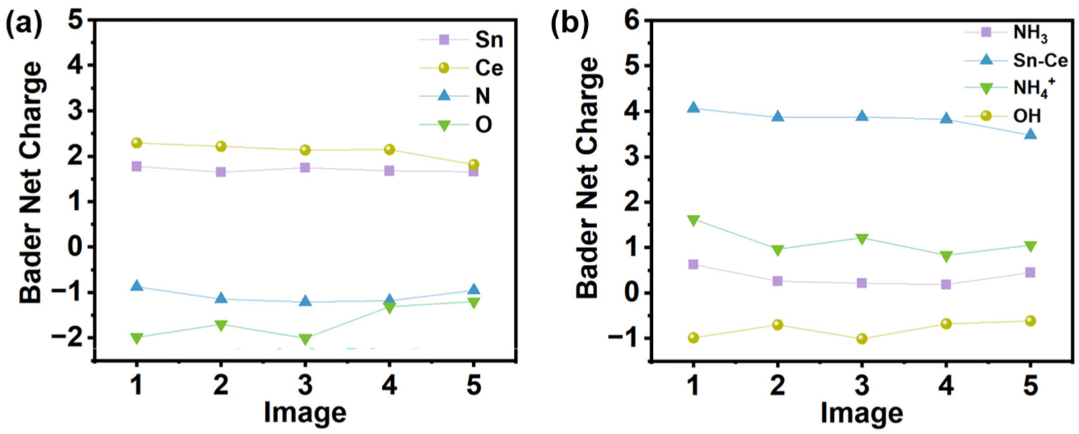

Figure S12. Bader net charges of (a) selected atoms and (b) selected molecular groups along the five-image water-mediated proton-transfer pathway in the hydrated PAN-Sn^{2+}-Ce^{3+} model.

Table S1. The composition of the sample.

Number of samples	Polymer matrix	Solvent	Metal additive components and molar ratio (Ce/Sn precursor molar ratio)
PAN	PAN	DMF	Pure PAN substrate (without metal additives)
Ce	PAN	DMF	1：0
Sn	PAN	DMF	0：1
Sn-$Ce_{10\%}$	PAN	DMF	1：9
Sn-$Ce_{30\%}$	PAN	DMF	3：7
Sn-$Ce_{50\%}$	PAN	DMF	1：1

Theoretical calculations

The first-principles calculations in this paper are all based on the density functional theory (DFT) framework and are completed using the Quantum ESPRESSO (QE) program package. The exchange-correlation effect is described using the generalized gradient approximation (GGA) under the Perdew-Burke-Ernzerhof (PBE) functional. The interaction between core electrons and valence electrons in the system is processed using projected augmented wave (PAW) pseudopotentials and ultra-soft pseudopotentials (USPP); elements such as C, N, O, and Ce are treated with PAW-type pseudopotentials, while H and Sn are treated with ultra-soft pseudopotentials.

This paper constructs a finite PAN short-chain model and places Sn^{2+}, Ce^{3+}, and NH_3/H_2O and other small molecules near the PAN cyanide group to study the coordination of metal ions, NH_3 adsorption, and proton migration processes. The finite PAN oligomer contains five –CH_2–CH(CN)– repeat units. The hydrated PAN–Sn^{2+}–Ce^{3+} model contains one H_2O molecule and one NH_3 molecule. The corresponding structural models contain 46 atoms with an elemental composition of $C_{15}H_{22}N_6OSnCe$. No explicit counterions are included in the models. All finite cluster models are placed in a cubic supercell of 26 Å × 26 Å × 26 Å to reduce the interaction between periodic images; at the same time, in the QE input, the isolated system correction (assume_isolated = 'mt') is further used to weaken the long-range electrostatic interaction of charged clusters. Since the studied system is a finite molecule/cluster model, the Brillouin zone integration is carried out at the Gamma point.

The plane-wave truncation energy of the valence electron wave function is set to 50 Ry, and the charge density truncation energy is set to 400 Ry. In charged systems containing Sn^{2+} and Ce^{3+}, the total charge is set according to the formal charge, and self-polarization calculation is used to describe the unpaired electron effect related to the metal ions. For systems involving metal ions, electron occupation adopts Gaussian smearing, with a broadening parameter of 0.01 Ry; for neutral molecules in closed shells or PAN reference systems, fixed occupation is used. The mixing parameter in the electron iteration is usually set to 0.3; for the difficult-to-converge Sn/Ce/H_2O/NH_3 composite system, a smaller mixing parameter is used and combined with the local Thomas-Fermi mixed mode to improve the convergence stability.

Geometric structure optimization uses the BFGS algorithm, optimizing only the internal

coordinates of the atoms while keeping the cell parameters unchanged. The electron self-consistent convergence threshold for the structure optimization stage is set to 1.0 × 10^{-6} Ry. Subsequently, based on the optimized structure, single-point SCF calculations are performed to obtain the binding energy, adsorption energy, reaction energy, and the electron density required for subsequent charge density analysis. Considering that this paper mainly focuses on the relative trends between different configurations, the single-point SCF calculation adopts an electron convergence threshold of 1.0 × 10^{-8} Ry.

In the differential charge density calculation, the complex and each fragment use exactly the same unit cell, atomic coordinate reference, and FFT grid to ensure that the cube files are strictly aligned in space. All charge density cube files are exported through the pp.x module of QE and further used for differential charge density visualization and planar average charge density analysis.

Bader charge analysis is based on the exported total charge density cube file. This paper defines the net Bader charge using the pseudopotential valence electron charge, that is:

$$q = Zval - Ne(Bader), \quad (S1)$$

Here, Zval represents the number of valence electrons of the corresponding pseudopotential, and Ne(Bader) is the electron number obtained by integrating within the Bader sub-region. Positive values indicate that the atom or group loses electrons, while negative values indicate that electrons are gained.

The proton migration energy barrier is calculated using the QE's NEB method. The NEB path consists of a total of 5 images. The initial state (IS) and final state (FS) are determined by the optimized reactant and product structures, and the intermediate images are obtained through linear interpolation. The path is optimized using the Broyden optimization scheme. According to the final converged NEB path, the highest energy point is located at internal image 3, corresponding to the proton migration saddle point.
